# FastSurfer-LIT: Lesion inpainting tool for whole-brain MRI segmentation with tumors, cavities, and abnormalities

**DOI:** 10.1162/imag_a_00446

**Published:** 2025-01-31

**Authors:** Clemens Pollak, David Kügler, Tobias Bauer, Theodor Rüber, Martin Reuter

**Affiliations:** AI in Medical Imaging, German Center for Neurodegenerative Diseases (DZNE), Bonn, Germany; Department of Neuroradiology, Bonn University Hospital, Bonn, Germany; Department of Epileptology, Bonn University Hospital, Bonn, Germany; Center for Medical Data Usability and Translation, University of Bonn, Bonn, Germany; A.A. Martinos Center for Biomedical Imaging, Massachusetts General Hospital, Boston, MA, United States; Department of Radiology, Harvard Medical School, Boston, MA, United States

**Keywords:** segmentation, lesion, tumor, software, inpainting, brain filling

## Abstract

Resection cavities, tumors, and other lesions can fundamentally alter brain structure and present as abnormalities in brain MRI. Specifically, quantifying subtle neuroanatomical changes in other, not directly affected regions of the brain is essential to assess the impact of tumors, surgery, chemo/radiotherapy, or drug treatments. However, only a limited number of solutions address this important task, while many standard analysis pipelines simply do not support abnormal brain images at all. In this paper, we present a method to perform sensitive neuroanatomical analysis of healthy brain regions in the presence of large lesions and cavities. Our approach called “FastSurfer Lesion Inpainting Tool” (FastSurfer-LIT) leverages the recently emerged Denoising Diffusion Probabilistic Models (DDPM) to fill lesion areas with healthy tissue that matches and extends the surrounding tissue. This enables subsequent processing with established MRI analysis methods such as the calculation of adjusted volume and surface measurements using FastSurfer or FreeSurfer. FastSurfer-LIT significantly outperforms previously proposed solutions on a large dataset of simulated brain tumors (N = 100) and synthetic multiple sclerosis lesions (N = 39) with improved Dice and Hausdorff measures, and also on a highly heterogeneous dataset with lesions and cavities in a manual assessment (N = 100). Finally, we demonstrate increased reliability to reproduce pre-operative cortical thickness estimates from corresponding post-operative temporo-mesial resection surgery MRIs. The method is publicly available at https://github.com/Deep-MI/LIT and will be integrated into the FastSurfer toolbox.

## 1 Introduction

**Fig. 1.**
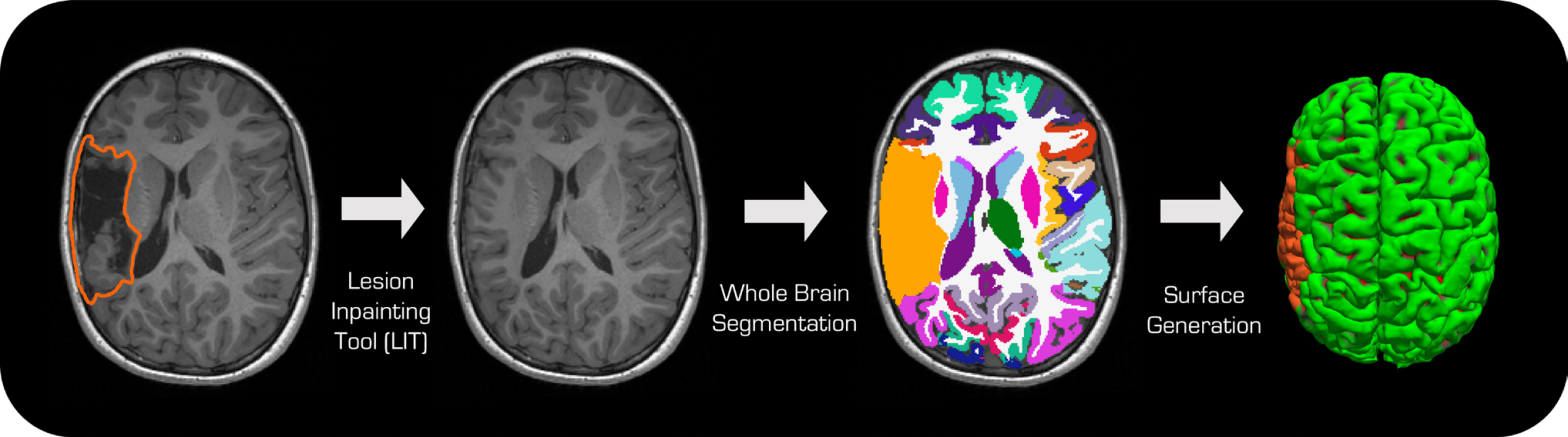
Overview of FastSurfer-LIT—Based on the two inputs (T1w-image and lesion mask, left), the FastSurfer-LIT pipeline synthesizes a realistic, lesion-free image (center left) by inpainting, then segments the brain into 79 structures (center right) and finally, reconstructs the pial and white matter surfaces (right). The lesion is in orange in segmentations and projected to the surface.

Neuromorphometry is a ubiquitous method for the analysis of brain MRI, used, for example, for the analysis of longitudinal changes during healthy aging or for group comparisons in clinical trials. While neuroimaging pipelines such as *FreeSurfer* (Fischl, 2012), *FSL* (Jenkinson et al., 2012), or *SPM* (Ashburner, 2009) can produce results for images with lesions (Radwan et al., 2021) (especially small ones), none of them are developed for, or validated on images with large lesions. In traditional atlas registration-based tools, such as FreeSurfer (Fischl, 2012), large errors in brain segmentation or premature termination can occur when abnormal changes in the brain tissue (lesions) make a registration with standardized templates challenging (Radwan et al., 2021). These failure modes persist in modern deep learning-based segmentation methods, which tend to perform poorly on data that have not been seen during training (out-of-distribution data). Even if the full brain segmentation is successful, the reconstruction of cortical surfaces is likely compromised when regions of the cortex are damaged, for example, due to invasive tumors or brain resection surgery (see Fig. 1). Meanwhile, the study of disease effects (e.g., glioblastoma effects on overall brain health) and the study of intervention (side-) effects (e.g., impact of radiotherapy, chemotherapy, or surgery) require morphometric analyses on brain MRIs with pathologies. Besides directly studying lesion effects, the blanket exclusion of images with abnormal structure can cause selection bias and decreased statistical power in downstream analysis of association studies. Even when the standard toolboxes complete without failure, the measurements on images containing lesions were found to be biased (Guo et al., 2019). To address this gap, researchers require specialized tools, which can generate accurate morphometric measurements in the presence of such pathologies and cavities.

In previous work, this challenge has been broadly addressed by replacing lesions with healthy looking tissue prior to processing. The process is commonly referred to as inpainting. Specifically, *SynthSR* (Iglesias et al., 2023) and *Virtual Brain Grafting (VBG)* (Radwan et al., 2021) have recently been proposed for inpainting healthy looking tissue in lesion areas. These significant advances enable previously challenging analyses, for example, of “personalized structural connectomics for moderate to severe traumatic brain injury” (Imms et al., 2023), “contralateral alterations in cortical morphology in patients with diffuse low-grade glioma” (Zhang et al., 2022), “structural plasticity of the contralesional hippocampus” (Liu et al., 2023), the role of the hippocampus in “recovery in persons with post stroke aphasia” (Schevenels et al., 2022), and “tracking the corticospinal tract in patients with high-grade glioma” (Zhylka et al., 2021). While VBG and SynthSR have unlocked volume and surface-based neuromorphometric analyses for these patient groups, we find that their application can sometimes be unstable, resulting in long runtimes, faulty segmentations, or unreliable estimates of cortical thickness. Additionally, previously proposed methods can only be used on images with a standard resolution of 1 mm, which requires lossy down-sampling of sub-millimeter MR images and results in decreased fidelity of segmentation, reconstruction, and subsequent analysis (Henschel et al., 2022). Other approaches, such as adding training cases with lesions to deep learning segmentation networks (Weiss et al., 2021), are currently limited, since no datasets with accurate whole-brain segmentations for patients with lesions exist. To cover the whole range of possible brain lesions, a very large and diverse dataset would be required, and even then, a newly trained network and fitting datasets would be required for every application. Robust inpainting, on the other hand, offers a general approach as it provides a synthetically corrected MRI that can be combined with various neuroimaging tools (e.g., also for surface reconstruction or registration purposes). For the *FastSurfer* (Faber et al., 2022; Henschel et al., 2020, 2022) and *FreeSurfer* (Fischl, 2012) toolboxes, for example, accurate inpainting enables both (i) whole-brain segmentation and (ii) cortical surface reconstruction, despite different underlying segmentation and surface reconstruction algorithms.

In this work, we propose *FastSurfer-LIT* a pipeline that performs whole-brain segmentation and surface reconstruction in the presence of small and large lesions and cavities on various resolutions, scanners, and types of lesion (see Fig. 1). The initial step of the pipeline is the lesion inpainting (LIT) network. To enable high-quality inpainting for arbitrary lesion shapes at multiple desired resolutions, we propose a novel resolution-independent Denoising Diffusion Probabilistic Model (DDPM). This network architecture enables inpainting for any shape or size of mask and can operate on multiple resolutions with high accuracy. By transferring spatial information through the reverse diffusion process, we generate high-quality plausible image content for masked areas, even when substantial parts of the brain are damaged. We combine LIT (the lesion inpainting tool) with FastSurfer (for whole-brain segmentation and surface reconstruction) into the FastSurfer-LIT neuroimaging pipeline and furthermore extend it with new post-processing tools to exclude abnormal regions from volumetric and surface statistical analysis to, for example, enable seamless group analysis including participants with brain lesions.

The evaluation of whole-brain segmentation in the presence of abnormalities is challenging, since no publicly available dataset with (manual) reference segmentations exists. Therefore, one of our experiments employs a tumor growth simulation (Subramanian et al., 2019) in combination with segmentations of lesion-free brains to generate a reference standard for our method. We deform the reference segmentations together with the image according to the tumor growth model to establish a dataset of 158 cases containing synthetic glioblastoma. Additionally, we also generate a second synthetic dataset of multiple sclerosis (MS) lesions (N = 39), by transferring lesions masks from patient cases to lesion-free MRI, which again provides us with pairs of reference segmentations and matching lesion masks. Besides these experiments on synthetic data, a blinded rater compared whole-brain segmentations of our method with VBG, the most promising competing method. This evaluation includes 100 cases of hospital patients with 14 different kinds of tumors and surgical cavities. Finally, we use FastSurfer-LIT to compare the consistency of cortical thickness estimates across 14 patients pre- and post-temporo-mesial resection surgery.

In summary, we contribute FastSurfer-LIT, a lesion inpainting, whole-brain segmentation and surface reconstruction pipeline, that

- processes brain MRI containing surgical cavities, tumors, and other lesions independent of their appearance, shape, or size when provided with a corresponding mask,
- performs inpainting natively on high-resolution images,
- outperforms state-of-the-art methods in whole-brain segmentations on MRI with synthetic lesions (and synthetic ground truth), and on MRI from hospital patients (using manual ratings),
- produces cortical thickness estimates with higher consistency across surgical intervention, compared with the state-of-the-art, and
- permits group comparisons to include cases with abnormalities (partially missing data).

These advancements are enabled by a resolution-independent DDPM with a novel slicing scheme for inference, which creates high-quality healthy looking brain MRI individualized for each case.

## 2 Related Work

### 2.1 Lesion inpainting methods

Instead of modifying every neuroimaging tool individually to make it robust—considering different lesion types, characteristics, and appearances—previous work has shown that replacing lesions with healthy looking tissue in the input image is an effective and widely applicable strategy. A straightforward approach for inpainting is to replace lesions with intensity values from neighboring areas (Battaglini et al., 2012; Chard et al., 2010; Griffanti et al., 2016; Guo et al., 2019; Magon et al., 2014; Popescu et al., 2014; Prados et al., 2016; Schmidt et al., 2019). Relying on the surroundings of a lesion works well to inpaint smaller areas, but for larger lesions, this approach falls short as whole structures and anatomical detail (e.g., cortical folds) are not recovered. Therefore, methods that work on larger lesions have to rely on a brain model to fill large areas with plausible brain structures. These models can either be learned or explicitly given by a healthy reference brain.

*Virtual Brain Grafting (VBG)* (Radwan et al., 2021) is a method that transfers healthy looking areas from a reference “donor brain” (Radwan et al., 2021) to a lesion affected area. First, the lesion area is specified by the user, then VBG’s 33-step pipeline transfers matching tissue from the healthy reference to the lesion area and uses FreeSurfer or FastSurfer for segmentation and surface reconstruction. Finally, segmentation maps containing the lesion and other lesion-specific outputs are generated by the pipeline. The initial inpainting process contains skull stripping, non-linear registration, and matching of noise and sharpness levels between the healthy reference and the lesioned brain. In conjunction, these steps often result in a seamless inpainting, which has unlocked the use of FastSurfer and FreeSurfer-based morphometrics for many applications (Imms et al., 2023; Liu et al., 2023; Schevenels et al., 2022; Zhang et al., 2022; Zhylka et al., 2021). Unfortunately, the registration-based transfer of healthy tissue is itself affected by the appearance of the lesion, which can make the inpainting less accurate for larger lesions (see, e.g., Appendix Fig. A3). The tissue from the template brain can also not be adjusted arbitrarily to the target brain, leading to potentially implausible inpaintings for uncommon brain structures. Some of these effects can be mitigated by hand-crafting templates for every population. This, however, is a time consuming process that requires new validation and quality control each time. Since competing methods do not need re-adjustment of templates, we have opted to use the standard template for all cases. Furthermore, in VBG, MRI are always standardized to a voxel resolution of 1 mm, creating interpolation artifacts and losing detail in sub-millimeter MRI, which is becoming widely available (Glasser et al., 2013; Henschel et al., 2022; Mellerio et al., 2014; Wattjes et al., 2006; Zaretskaya et al., 2018). Finally, the high complexity of the pipeline introduces significant computational costs and adds more than 2.5 hours to the runtime of the FreeSurfer and FastSurfer pipelines, corresponding to a 64% and 192% increase, respectively (see Section 4.4).

SynthSR (Iglesias et al., 2023), on the other hand, is a deep learning model, which prepares images for processing with FreeSurfer based on the previously seen training data. The method invokes only a single Convolutional Neural Network (CNN) inference resulting in run times in the order of seconds for inpainting. The training data consist of a combination of T1-weighted scans from the OASIS (Marcus et al., 2007) dataset as a target and synthetic MRI generated to imitate the appearance of MRI with various resolutions and modalities as input scans. SynthSR standardizes the image, which includes the inpainting of lesions, but also a change of image contrast, orientation, and voxel resolution to 1 mm. This standardization enables use of FreeSurfer with previously unusable clinical images, such as low-resolution, anisotropic voxels, or CT images, as well as some images with lesions. In our experiments we observed that the inpainting of larger lesions is less reliable than smaller lesions (see Appendix Fig. A3). In contrast to other inpainting methods, SynthSR does not require a lesion mask. While this may seem like a significant advantage, it also removes the guarantee that synthetic intensity changes are limited to the lesion area. Therefore, image changes can occur in unexpected areas (see Appendix Fig. A2.B,C). It also means that the neural network input includes the lesion itself, making it dependent on the lesion appearance and, thus, more prone to failure for out-of-distribution data. Ultimately, a lesion mask is still required to exclude the synthesized region from downstream analysis.

In addition to SynthSR, various other deep-learning methods for inpainting MR images have been proposed. They are, however, developed for the inpainting of specific target abnormalities (e.g., removing MS-lesions (Clèrigues et al., 2023; Tang et al., 2021), removing artifacts (Xie et al., 2023), re-facing (Xiao et al., 2022), atlas reconstruction (Xing et al., 2022), and synthetic validation of images with atrophy (J. Wang et al., 2023)). The aforementioned methods use *Generative Adversarial Networks (GAN)*, which were trained by removing areas shaped like the target abnormality from the training images (making them independent of its appearance). The adversarial training paradigm requires a second network to assess the realism of images. A major limitation of GANs is that they are not expected to generalize well to previously unseen mask shapes (Lugmayr et al., 2022), which means that lesions of unseen size or shape cannot be accurately replaced by these specific methods.

Recently, *Denoising Diffusion Probabilistic Models (DDPM)* have emerged as the state-of-the-art method for natural image inpainting (Lugmayr et al., 2022). Besides superior performance compared with GANs (Dhariwal & Nichol, 2021; Lugmayr et al., 2022), these deep learning models can be trained by de-noising images (also called the “reverse diffusion” process), which removes the necessity to generate masks for inpainting during training. During inference, the reverse diffusion process can be leveraged to generate plausible inpainted regions for arbitrary masks, which was previously challenging with GANs (Lugmayr et al., 2022). They are also independent of the appearance of the inpainted region. DDPMs have now been expanded to 2.5D for 3D inverse problems, such as 3D MRI and CT reconstruction (Chung et al., 2023; Lee et al., 2023) by applying two 2D diffusion models in two orientations of the volume. This process is similar to view aggregation of 2.5D segmentation models (Henschel et al., 2020; Roy et al., 2022) and creates coherent volumes during the reverse diffusion process. Because of their strong theoretical advantages and proven utility, we use a DDPM for inpainting in our method. Finally, no resolution-independent DDPM method has been introduced so far creating a gap for sub-millimeter MR acquisition protocols.

### 2.2 Evaluation of whole-brain segmentation in the presence of large lesions

A general challenge for the development and evaluation of lesion-robust segmentation is the missing ground truth data. While large datasets with manual lesion segmentations exist (Aerts & Marinazzo, 2018; Aerts et al., 2020; Baid et al., 2021; Bakas et al., 2022; Menze et al., 2015), no database combining lesion segmentations with (manual) whole-brain segmentation has been published to date. Therefore, the authors of VBG evaluated their method based on two datasets:

1. Patient dataset: 10 patients with glioma.
2. In the absence of ground truth labels, the quality of segmentations of the patient dataset was assessed by experts.
3. Synthetic dataset: 100 synthetic cases from 10 gliomas inserted into 10 “healthy” brain volumes.
  The mass effect was mimicked by nonlinear registration of the images. Reference segmentations were generated with FreeSurfer based on deformed images (without lesion insertion).

These two analyses give a good overview of the method performance with reference-based measures such as Dice Similarity Coefficients and expert ratings. However, the patient dataset was limited in size (10) and diversity (only glioma lesions). For the synthetic dataset, the reference standard was generated by FreeSurfer after deformation by the synthetic mass effect, which could lead to decreased quality in the FreeSurfer outputs used as ground truth.

SynthSR was evaluated on three datasets containing lesions:

1. ATLAS (Liew et al., 2018): 655 volumes of stroke patients.
2. BraTS (Menze et al., 2015): 1251 volumes of glioblastoma patients.
3. BraTS-Registration (Baheti et al., 2021): 140 volumes of glioma patients.

While the comparison on these datasets did not include a baseline method, an analysis for the ATLAS dataset of the ipsi- and contralateral volumes of hippocampus, amygdala, thalamus, putamen, and caudate shows asymmetry patterns consistent with the literature. For the lesion inpainting, this was the only analysis of brain segmentations. Additionally, SynthSR was used to aid in the creation of a brain atlas for the BRaTS dataset, showing spatial distribution of gliomas consistent with the literature. On the BraTS-Registration dataset, the combination of SynthSR with NiftyReg (Modat et al., 2014) reduced the average landmark error.

## 3 Materials and Methods

### 3.1 Data

For the training and evaluation of our method, we compile four meta-datasets:

1. No-lesion dataset: To learn the anatomy of brains without large lesions, we combine 11 publicly available datasets into a heterogeneous multi-resolution dataset of 1750 volumes with isotropic resolutions of 0.7, 0.8, 0.9, and 1 mm. To prevent overoptimistic results by leakage of information from the final test set into the training procedure, we use the same training, validation, and test splits used for the development of FastSurfer (Henschel et al., 2022), resulting in (1315, 80, 355) volumes in the (training, validation, test) set:
  (a) HCP (Glasser et al., 2013) (30, 20, 80)
  (b) RS (Breteler et al., 2014) (30, 20, 80)
  (c) ABIDE-I (Di Martino et al., 2014) (68, 0, 20)
  (d) ABIDE-II (Di Martino et al., 2017) (0, 0, 25)
  (e) ADNI (Jack et al., 2008) (215, 8, 40)
  (f) IXI (“IXI – Information eXtraction from Images”, n.d. (400, 0, 43)
  (g) LA5C (Poldrack et al., 2016) (203, 9, 15)
  (h) MBB (Babayan et al., 2019) (195, 0, 0)
  (i) MIRIAD (Malone et al., 2013) (30, 7, 0)
  (j) OASIS1 (Marcus et al., 2007) (79, 11, 35)
  (k) OASIS2 (Marcus et al., 2010) (65, 5, 17)
    To reduce redundancy, details on these datasets can be found in the FastSurferVINN paper (Henschel et al., 2022), where this meta-dataset was first introduced.
2. Synthetic glioblastoma dataset: We use a tumor growth simulation (Subramanian et al., 2019) to generate tumor areas and plausible deformations for 58 randomly selected cases from the healthy validation dataset and 100 from the healthy test set. We detail the simulation process in Section 3.2.
3. Synthetic MS lesion dataset: We use a publicly available dataset of multiple sclerosis (MS) lesions (Commowick et al., 2018, 2021)^*^ and transfer the lesion masks from the original MRI to 39 cases of the healthy test set.
4. Patient dataset: We obtain three datasets with lesion-afflicted images and lesion masks to evaluate our method on clinical cases.
  (a) UPENN-GBM (Bakas et al., 2021, 2022; Clark et al., 2013): 630 MRI from glioblastoma patients “acquired during routine clinical practice, at the University of Pennsylvania Health System” (Bakas et al., 2022). Images are acquired with multiple scanners, MR sequences, field strengths, and voxel sizes from 0.9 to 5 mm anisotropic. For our analysis, we randomly select 150 isotropic images.
  (b) BTC (Aerts & Marinazzo, 2018; Aerts et al., 2018, 2020): Overall 44 MRI acquired at the Ghent University Hospital, Belgium. The dataset includes volumes of 11 patients with glioma and 14 patients with meningioma before surgery and 7 and 12 follow-up post-operative scans respectively, all with a voxel size of 1 mm.
  (c) UKB: 76 MRI of patients acquired at the University Clinic of Bonn, Germany with 11 different types of lesions including hippocampal resections, porencephaly, and Rasmussen’s encephalitis acquired at 0.8 mm voxel size.
5. Temporo-mesial resection surgery dataset: 15 pairs of pre- and post-operative scans of patients with mesial temporal lobe epilepsy undergoing temporo-mesial resection surgery acquired at the University Clinic of Bonn, Germany with 0.8 mm voxel size.

Participants of the individual studies gave informed consent in accordance with the Institutional Review Board at each of the participating sites. Complete ethic statements are available at the respective study web pages and cited publications.

### 3.2 Tumor growth simulation

The lack of manual whole-brain segmentations for images with lesions creates a significant obstacle for quantitative evaluation. To overcome this, we generate MRI with synthetic glioblastoma and accurate segmentation labels by augmenting the validation and test sets of the *no-lesion dataset* with a tumor growth simulation model (Subramanian et al., 2019).

The simulation by Subramanian et al. was shown to create large, realistic deformations mimicking those of real glioblastoma closely (Subramanian et al., 2019). Its inputs are segmentations for gray matter, white matter, cerebrospinal fluid, and ventricles as well as an initial starting point for tumor growth. Required segmentations are generated by FastSurfer based on the no-lesion MRI. The starting point is chosen randomly within the brain mask. For implausible locations, tumor growth is typically minimal and such cases are subsequently excluded. The growth model provides a tumor mask and a deformation warp field mimicking the tumors mass effect. We use the deformations to propagate the reliable whole-brain segmentations created from images without lesions to the images with tumor mask and mass effect. Due to the high computational cost of growth simulation, we limit the synthetic dataset to a subset of (58, 100) of the no-lesion (validation, test) sets. Importantly, we ensure there is no overlap between data used for method development (i.e., training and validation) and the test of FastSurfer, our LIT network, and the FastSurfer-LIT pipeline.

### 3.3 Synthetic MS lesion

Similar to synthetic glioblastoma, we also generate a dataset with synthetic MS lesions for the validation of our method. In this case, the mass effect of lesions is negligible, however, the pattern and distribution of lesions in the brain are unique for each individual. Therefore, we map existing lesion masks onto the no-lesion test set via non-linear registration (ANTsPy version 0.5.4 SyN (Avants et al., 2008)). With this strategy, we follow evaluations previously performed for MS-specific inpainting tools (Clèrigues et al., 2023; Tang et al., 2021). We use the resulting 39 cases with synthetic MS lesion masks only for the final evaluation of our method and not during development.

### 3.4 Diffusion model for inpainting

At the core of our method is the LIT inpainting module which follows the established approach of replacing anomalous areas with healthy looking tissue prior to segmentation and surface reconstruction. Contrary to competing methods (Iglesias et al., 2023; Radwan et al., 2021), we aim to leave regions outside of the marked lesion area completely unmodified. Therefore, we keep image intensities outside of the tumor mask unchanged and also do not alter the image resolution.

**Appendix Fig. A1.**
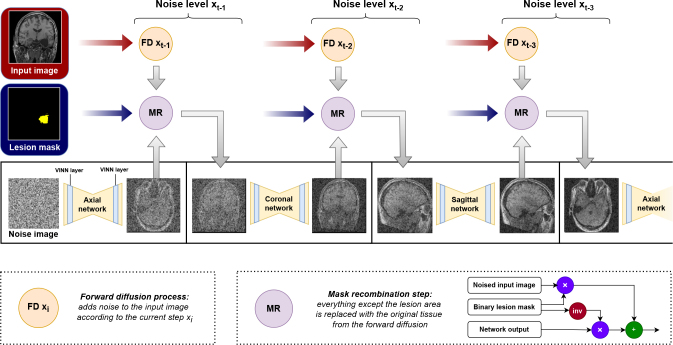
Overview of the DDPM inpainting process. The architecture for the axial, coronal, and sagittal networks is shared, but weights differ. Mask recombination is used to guide the inpainting process. Denoising per step is exaggerated in the figure for better viewability. Not shown are slab selection and slab-wise denoising.

We propose a resolution-independent DDPM architecture that can accurately generate images on multiple resolutions (isotropic) via the reverse diffusion process. We base our approach on latent diffusion models (LDM) (Pinaya et al., 2022, 2023; Rombach et al., 2022) competent in generating high-resolution natural images (Rombach et al., 2022) and brain MRI (Pinaya et al., 2023) (for an overview of the general data flow in DDPM inpainting, see Appendix A: Appendix Fig. A1). Contrary to the usual U-Net-like architecture, with only fixed up- and down-sampling by factor 2, we replace one set of up- and down-sampling with VINN (Voxel-size Independent Neural Network) layers. These neural network layers adapt the up- and down-sampling based on the input resolution to standardize the size of feature maps in the latent space. This reduces the voxel-size variance—leading to more effective learning for multi-resolution data.

The vast image size of high-resolution images beyond $256^{3}$ voxels makes the use of fully 3D neural networks impractical (Roy et al., 2022). However, inpainting large abnormalities likely benefits from context information of the whole brain. The intact contralateral hemisphere, for example, may contain important priors for matching healthy tissue (Xing et al., 2022). In fact, even the background noise may provide important information about the brain’s appearance in the MRI (Pollak, Kügler, Breteler, et al., 2023; Pollak, Kügler, & Reuter, 2023). To use an extended context, while keeping the network and its memory requirements manageable, we combine two strategies: (1) Using three separately trained 2D models for each of the anatomical planes (multi-view) and (2) choosing shifted slabs during each call of the 2D inference with different offsets orthogonal to the current view (varying spatial context). The central idea for both of these strategies is to propagate spatial information via the iterative diffusion process by providing changing information during each network inference. More specifically, our LIT method rotates between using axial, coronal, and sagittal views, similar to Lee et al. (2023). In contrast to Lee et al. (2023), we use slabs of seven neighboring image slices (with full image height and width) as network input, instead of only a single slice. Then we select slabs with different offsets to provide new context for every inference step. While still compatible with the 2D networks, slabs provide additional information in the direction orthogonal to the current view.

Our modifications to the standard DDPM inference scheme and architecture do not require additional computational resources during inference. Furthermore, we only perform inference for slabs containing the lesion area (Lugmayr et al., 2022), which linearly reduces inference cost with lesion size. Overall, the LIT DDPM is an accurate, efficient inpainting method, which natively supports multiple resolutions, uses high spatial context, and is completely independent of the target region’s shape or appearance.

### 3.5 Whole-brain segmentation and surface reconstruction

For the segmentation and surface reconstruction after inpainting, we employ two popular toolboxes: FastSurfer and FreeSurfer. FastSurfer (version 2.2.0) uses a voxel size independent neural network (VINN) (Henschel et al., 2022) for whole-brain segmentation, while FreeSurfer (Fischl, 2012) (version 7.4.1) is based on a probabilistic atlas segmentation. Both tools provide subsequent white matter and pial surface reconstructions. We further extend FastSurfer and FreeSurfer with functionality to handle lesion areas during statistical analysis of morphometric estimates. Segmentations of the inpainted images are modified retrospectively by replacing them within the inpainted area with a specific lesion mask label. This segmentation is then mapped to the vertices of the pial and white surfaces, by (i) dilating the lesion mask, (ii) marking the vertices that are intersecting with the surface, and (iii) smoothing the mask border and filling holes on the surface (mode filter). The vertices labeled as lesion mask can then be ignored in downstream statistical group analyses on the participant level (as demonstrated in Section 4.8). This permits statistical analysis even for areas, where lesions are present for some cases in a dataset. While we directly implement LIT into the FastSurfer pipeline, it can additionally be used as a standalone, general inpainting tool and combined with other neuroimaging software.

### 3.6 Evaluation metrics

We extensively validate FastSurfer-LIT using a variety of metrics for inpainting, segmentation, surface reconstruction, and computational efficiency.

#### 3.6.1 Perceptual similarity

To compare the effect of our modifications to the standard DDPM model, we perform inpainting on the no-lesion dataset using simulated masks and use common perceptual quality metrics to test whether our approach can re-generate the masked areas. First, the Structural Similarity Index Measure (SSIM) (Z. Wang et al., 2004)

$$SSIM\left(x,y\right)=\frac{\left(2\mu_{x}\mu_{y}+c_{1}\right)\left(2\sigma_{xy}+c_{2}\right)}{\left(\mu_{x}^{2}+\mu_{y}^{2}+c_{1}\right)\left(\sigma_{x}^{2}+\sigma_{y}^{2}+c_{2}\right)}$$ (1)

“compares local patterns of pixel intensities that have been normalized for luminance and contrast” (Z. Wang et al., 2004). Here $\mu_{x}$ and $\mu_{y}$ are the mean of x and y, $\sigma_{x}^{2}$ and $\sigma_{y}^{2}$ are the variance of x and y, $\sigma_{xy}$ is the covariance of x and y, finally $c_{1}={\left(0.01*L\right)}^{2}$, $c_{2}={\left(0.03*L\right)}^{2}$, where L is the dynamic range. To calculate the SSIM, we use PyTorch’s ignite framework (Fomin et al., 2020) with a Gaussian kernel of size 11 and standard deviation of 1.5. The SSIM quantifies the similarity of two image areas between -1 and 1, where 1 is a perfect match, 0 indicates no similarity, and -1 would refer to inverse correlation.

As a second metric, we choose the peak-signal-to-noise ratio (PSNR)

$$PSNR\left(x,y\right)=10\cdot {\text{log}}_{10}\left(\frac{\text{M}}{MSE\left(x,y\right)}\right),$$ (2)

where M is the maximum value in the image representation (255 in our case) and MSE is the mean squared error. We also use the ignite framework to calculate this metric. PSNR quantifies the similarity of two image areas x, y in decibel (dB), where higher values indicate a higher similarity.

#### 3.6.2 Segmentation quality

To compare segmentations of the tested method with a reference standard, we use the Dice Similarity Coefficient (DSC) and the Hausdorff Distance (HD). The DSC is defined as

$$DSC\left(X,Y\right)=\frac{2\left|X\cap Y\right|}{\left|X\right|+\left|Y\right|}.$$ (3)

This metrics show the agreement of two binary masks X and Y. In our case, these masks indicate the location and extent of brain structures. The DSC is zero when there is no overlap between prediction and reference standard. A perfect agreement is indicated by a DSC of 1. The HD is defined as

$$HD\left(X,Y\right)=\text{max}\left\{\underset{x\in X}{\text{sup}}d\left(x,Y\right),\underset{y\in Y}{\text{sup}}d\left(X,y\right)\right\}.$$ (4)

For the same binary masks X and Y, the HD describes whether the edges of the two structures are close to each other. To get a more robust metric, we do not choose the furthest possible distance, but the 95th percentile. We always indicate the HD in millimeters, where 0 mm means that there is a perfect match of structures up to the 95th percentile. To show whether one method outperforms another significantly, we use the Wilcoxon rank-sum statistic implemented in the SciPy (Virtanen et al., 2020) library. The null hypothesis for this test is that the method ranking is random.

#### 3.6.3 Method failures, runtime, and topological defects

In addition to the reference-based evaluations from previous sections, we analyze three per-run meta-data metrics: success rate, overall runtime, and the surface defect count. Large lesions can cause whole-brain segmentation or surface reconstruction pipelines to crash or fail to produce meaningful outputs. While unsuccessful runs do not affect analysis directly, they can introduce selection biases into downstream analysis. Therefore, we always exclude cases with any failures for any methods (except otherwise stated).

To determine the success rate, we track the runtime of pipelines and terminate instances that do not generate all required outputs within 24 hours. We run this benchmark for a subset of 10 cases of the patient dataset on a dedicated desktop workstation (Intel Xeon W-2245, 64GB RAM, Nvidia Quadro RTX 4000 8GB, solid state drive). We do not use parallel processing or use the machine for other tasks, to avoid interaction effects. Additionally, we report the overall runtime and runtime of successful cases, since the runtime itself is a valuable criteria due to the associated wait times and energy costs.

Finally, we noticed that faulty or low-quality surface reconstruction is a typical failure mode for all compared pipelines. Such surface errors lead to topological surface defects, pipeline crashes, and long pipeline run times. The number of surface defects is also a common quality measure for quality control of FastSurfer and FreeSurfer runs (Esteban et al., 2017; Rosen et al., 2018). Therefore, we also report the average surface defects on the larger synthetic lesion dataset.

#### 3.6.4 Manual comparison of whole-brain segmentation

In a visual validation, a domain expert compares segmentations derived from the two best-performing inpainting methods: VBG and LIT. This analysis is performed separately when using FreeSurfer or FastSurfer as the segmentation tool. The expert selects the superior segmentation map based on subcortical regions and gray/white matter (GM/WM) boundary for each shown case, while ordering of cases and methods in the viewer is randomized. Additionally, the rater also marks cases as “failure” if multiple major errors occurred, such as missing entire sulci or gyri, mislabeling of the cerebellum as cortex, or completely misplaced corpus callosum.

We randomly draw 50 cases from the patient dataset, choosing from a “random” subset and a “high difference” subset of cases. The high difference cases are selected based on large differences between VBG and LIT-based segmentation maps, which are expected to be more challenging and thus suitable for method comparison (Isensee et al., 2024). More specifically, the cases are selected according to the highest peak value on the Gaussian smoothed segmentation difference map. Since all cases are specific to the used method, these sets differ between FastSurfer and FreeSurfer, and can, therefore, only be used for a direct comparison across inpainting approaches within each segmentation method.

To support the visual inspection during these tasks, we develop a custom rating tool, which guides raters through the process, collects inputs, selects and highlights an area of interest, highlights differences of segmentation maps, etc. (see Appendix C, Appendix Fig. A5). During all rating, a free text field for comments captures the reasoning and supports retrospective analysis of rating decisions. To assess the statistical significance of binary rater decisions (e.g., “Which of two methods is better?”, “Did a method fail?”), we use Fishers exact test (Fisher, 1992), implemented in the SciPy software library (Virtanen et al., 2020).

#### 3.6.5 Consistency of cortical thickness estimates pre- and post-surgery

Finally, we test the consistency of cortical thickness estimates pre- and post-temporo-mesial resection surgery for MRI of patients with epilepsy. Pre-surgery segmentations and surfaces can be generated by the standard neuroimaging pipelines, as existing pipelines are sufficiently robust to temporal lobe atrophy, while post-surgery images require lesion inpainting.

For this test, we smooth all cortical thickness maps (full width at half maximum 15) and map the surfaces onto a common template (fsaverage). Then, we calculate the intraclass correlation coefficient (ICC) (McGraw & Wong, 1996) to determine thickness similarity in the paired samples of pre- and post-surgery images on a vertex level. In this case, ICC indicates the similarity of the thickness values on the surface, where 1 indicates perfect reproducibility. Note, that we use the degree of absolute agreement among measurements (criterion-referenced reliability), which compares equality, not only correlation.

## 4 Results

In the following section, we first present the effect of our modifications to the standard DDPM inference and architecture. Then, we jointly evaluate our LIT DDPM in two scenarios: (a) inpainting with the FastSurfer pipeline for inpainting (FastSurfer-LIT) and (b) inpainting combined with FreeSurfer for segmentation and surface reconstruction on the same set of experiments. In each scenario, the relevant reference is generated based on lesion-free images by FastSurfer or FreeSurfer, respectively. Additionally, we evaluate multiple method combinations with real-world images, where no reference segmentations are available.

### 4.1 Method ablation

Initially, we evaluate the effect of our modifications to the baseline 2D DDPM architecture and inference scheme, iteratively removing components of our method and re-evaluating—until only the baseline method remains (ablation study). While we change the network architecture and inference scheme, the number of network parameters and the required inference steps and input sizes (slabs) are the same across variants. This experiment is performed on the validation split of the synthetic lesion dataset used for method development. First, we run the DDPM-based inpainting in conjunction with the FastSurfer segmentation (Henschel et al., 2022). Then, we calculate SSIM and PSNR to judge the inpainting quality, as well as DSC and HD95 for segmentation performance. Note that the segmentation method is fixed and only the inpainting changes, resulting in different segmentation accuracy. To reduce computational cost during method development, we omit surface reconstruction and generate segmentation maps with the FastSurferVINN neural network only.

The results are shown in Table 1, where the LIT inpainting outperforms other DDPM variants. Since the synthetic lesion only affects the FastSurferVINN inference on volume slices intersecting the lesion, segmentations are identical in most of the volume. In consequence, most structures of the segmentation are not affected and differences of average DSC and HD appear small. Implausible inpainting can, however, have strong impact on the affected slices, and also cause compounding effects further downsteam in the processing of FastSurfer and FreeSurfer.

**Table 1.** Method ablation results.

Method configuration	PSNR [dB] ↑	SSIM ↑	DICE ↑	HD95 [mm]↓
**LIT** (VINN-DDPM with VA & VSC) (proposed method) VINN-DDPM with VA DDPM with VA Baseline (2D DDPM)	**29.64** 29.30 29.52 27.91	**0.72** 0.70 **0.72** 0.66	**0.9492** 0.9491 0.9484 0.9482	**0.6217** 0.6236 0.6595 0.6640

All scores are calculated on the validation set with synthetic lesions and mass effects. Segmentation differences based on FastSurfer appear small, since segmentations perfectly match on slices unaffected by the lesion. Bold values identify the best performing method. LIT is bold to highlight the “final method”.

LIT = Lesion Inpainting Tool (proposed method), VA = view aggregation, VSC = varying spatial context, DDPM = Denoising Diffusion Probabilistic Model, VINN = Voxel Size Independent Neural Network, PSNR = Peak Signal-to-Noise Ratio, SSIM = Structural Similarity Metric, DICE = Dice similarity coefficient, HD95 = 95th percentile Hausdorff distance.

### 4.2 Synthetic glioblastoma data

**Fig. 2.**
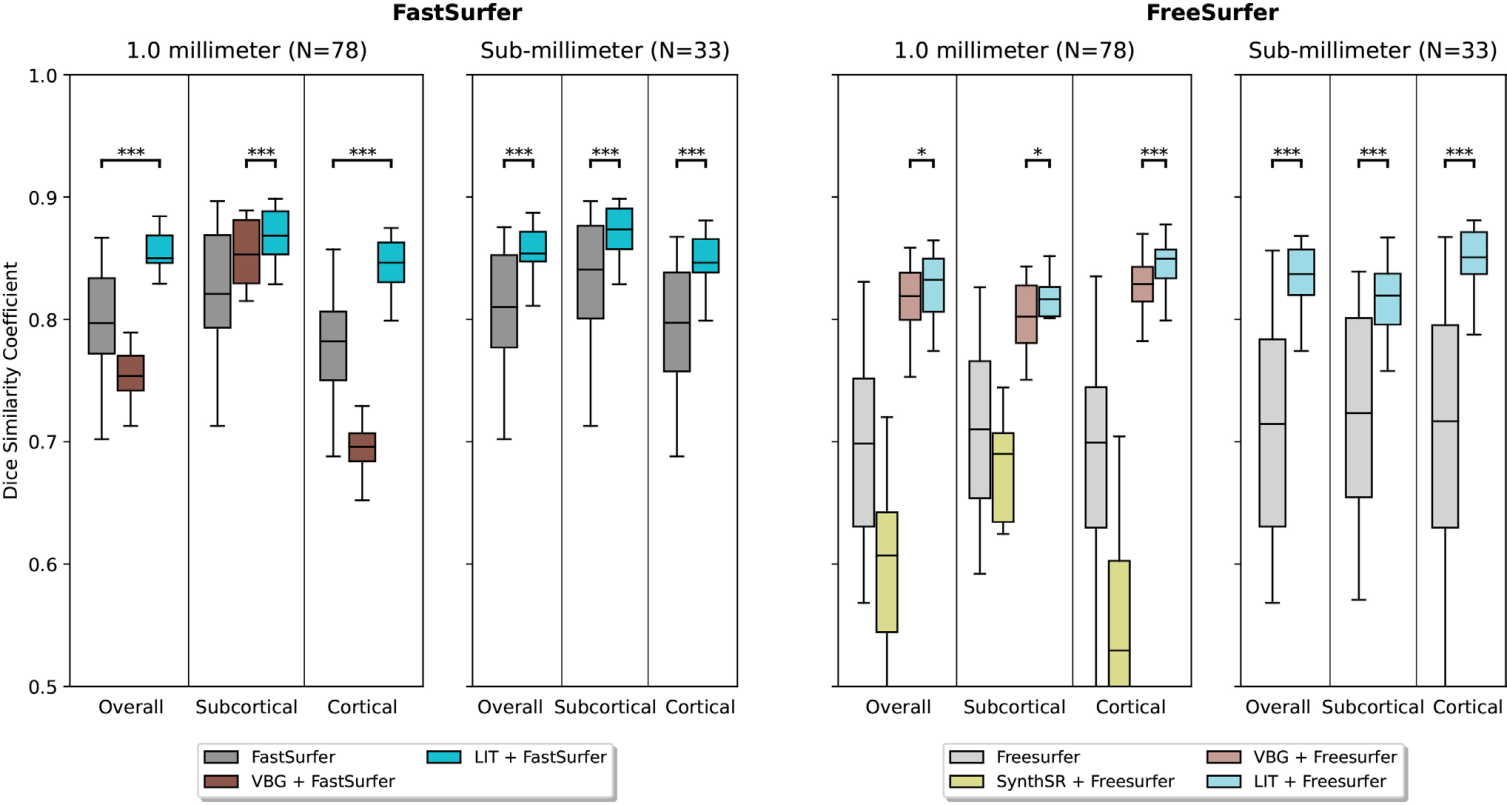
Method comparison on volumes with simulated glioblastoma. Reference segmentations are generated from lesion-free images by FastSurfer (left) and FreeSurfer (right), respectively (see Section 3.2). Our LIT inpainting significantly outperforms the state-of-the-art on all tests, measured by the Wilcoxon rank-sum statistic. *p*-Values are indicated for comparison with the second best method. (*p<0.05 , **p<0.005 , ***p<0.0005 ).

We evaluate the accuracy of cortical and sub-cortical segmentations on the synthetic glioblastoma dataset, which contains deformed FreeSurfer and FastSurfer segmentations as a reference standard (see Section 3.2). In the first scenario (using FastSurfer), we compare segmentation performance for (i) no inpainting (baseline) and the two inpainting methods (ii) VBG, and (iii) LIT (ours) (see Fig. 2, left). Both LIT and VBG are developed for compatibility with both FreeSurfer and FastSurfer. We exclude SynthSR, since it has only been evaluated with FreeSurfer previously and often fails in conjunction with FastSurfer (see Section 4.4). LIT significantly outperforms the state-of-the-art methods on subcortical, cortical, and average DSC. VBG consistently leads to lower scores on cortical DSC, when used together with FastSurfer even performing worse than the unmodified FastSurfer. Mean Hausdorff Distances (HD) of segmentations show the same trends as DSC (no inpainting: 2.16 mm, VBG: 2.69 mm, LIT: 1.34 mm) with all differences of LIT to the second best method significant (p<0.0005 ). Additionally, we show the segmentation accuracy on volumes with resolution 0.7 mm, 0.8 mm, and 0.9 mm. Here, the only available baseline to compare our FastSurfer-LIT pipeline with is the no inpainting baseline, since the competing methods can only produce segmentations at 1 mm voxel resolution. Our method provides consistent results on sub-millimeter resolution for both DSC and HD (no inpainting: 1.98 mm, LIT: 1.14 mm), indicating robust generalization to high-resolution inpainting and segmentation.

The second scenario illustrated in Figure 2b swaps the FastSurfer pipeline for reference generation and segmentation with FreeSurfer to show the segmentation performance of LIT in a different setting. Here, we compare the effect of (i) no inpainting (baseline), (ii) SynthSR, (iii) VBG, and (iv) LIT (ours) with FreeSurfer segmentations on the same images with synthetic glioblastoma. Our method outperforms the state-of-the-art methods with statistical significance across the different structures and resolutions. VBG reaches good performance on subcortical DSC and on cortical DSC. SynthSR under-performs FreeSurfer without any modifications on sub-cortical and cortical regions, which might stem from SynthSR modifying and standardizing the contrast of the whole image. The evaluation of Hausdorff Distances (HD) paints a similar picture (no inpainting: 3.27 mm, SynthSR: 4.47 mm, VBG: 1.42 mm, LIT: 1.33 mm). Differences of LIT to the second best method are also statistically significant (p<0.0005 ). For volumes with sub-millimeter resolution, our method also outperforms the FreeSurfer-only baseline in DSC and HD (no inpainting: 1.93 mm, LIT: 1.13 mm).

### 4.3 Synthetic multiple sclerosis data

**Fig. 3.**
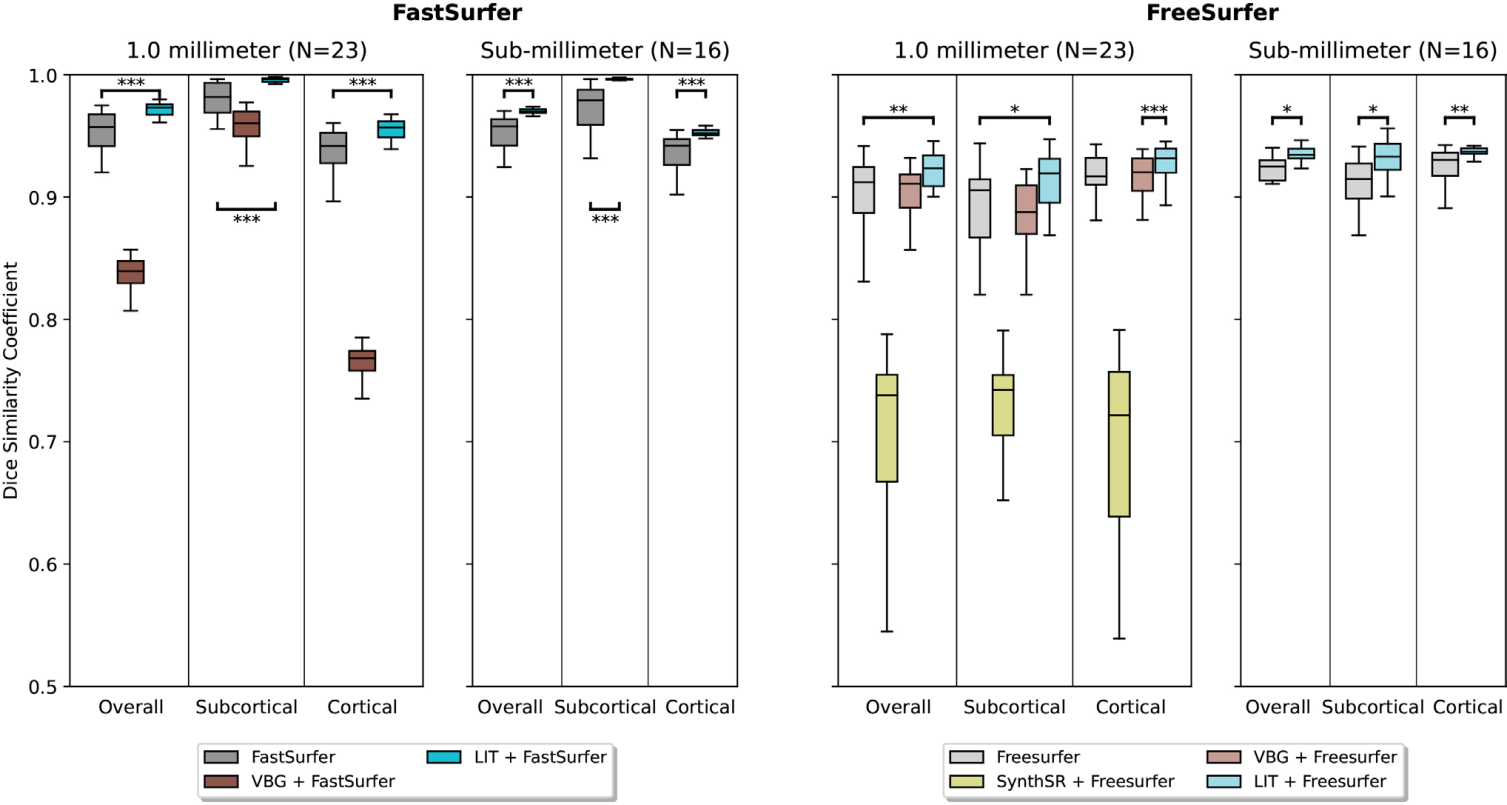
Method comparison on images with synthetic multiple sclerosis lesions. Reference segmentations are generated from lesion-free images by FastSurfer (left) and FreeSurfer (right), respectively (see Section 3.3). Our LIT inpainting significantly outperforms the state-of-the-art on all tests, measured by the Wilcoxon rank-sum statistic. *p*-Values are indicated for comparison with the second best method. (*p<0.05 , **p<0.005 , ***p<0.0005 ).

We repeat the analysis of the previous section with a new dataset of MRI with synthetically generated multiple sclerosis lesions. A notable difference between the two datasets is that the synthetic glioblastoma cases include a simulated mass effect, which jointly perturbs images and reference segmentations. This is absent for the synthetic MS lesions, making it a more direct measure of inpainting accuracy, and should yield to higher scores across the board. Additionally, MS lesions are typically smaller than the simulated glioblastoma, but can be challenging since many lesions can be present in an image and they may be distributed throughout multiple brain regions. In the first scenario (Fig. 3, left), using FastSurfer, with (i) no inpainting, (ii) VBG, and (iii) LIT, our method (LIT) significantly outperforms the two others on 1.0 mm. The same is true for sub-millimeter data, where the (i) no inpainting baseline is the only available comparison. Independent of resolution, our method achieves DSC close to 1 for subcortical structures, indicating supreme inpainting performance. Mean Hausdorff Distances (HD) of segmentations show the same trends as DSC of 1 mm resolution volumes (no inpainting: 0.85 mm, VBG: 2.42 mm, LIT: 0.61 mm) and sub-millimeter volumes (no inpainting: 0.74 mm, LIT: 0.49 mm) with all differences of LIT to the second best method significant (p<0.0005 ).

For the second scenario, using FreeSurfer, LIT also significantly outperforms the (i) no-inpainting baseline and the competing methods (ii) SynthSR and (iii) VBG, on both 1.0 mm and sub-millimeter MRI. Mean Hausdorff Distances (HD) of segmentations show the same trends for both 1.0 mm resolution (no inpainting: 1.25 mm, VBG: 1.28 mm, SynthSR: 3.02 mm, LIT: 1.14 mm) and sub-millimeter resolution (no inpainting: 1.04 mm, LIT: 0.91 mm). The difference in HD of LIT to the second best method (FreeSurfer) is significant for 1.0 mm resolution volumes (p<0.05 ), but not quite for sub-millimeter resolution volumes (p=0.057 ). We note that inpainting with VBG does not improve segmentation, compared with the no-inpainting baseline, except for cortical regions on 1.0 mm MRI processed with FreeSurfer. VBG has previously only been evaluated on glioma and gliomatous lesions. Our results on synthetic MS lesions indicate that inpainting multiple disconnected regions may be outside of VBGs area of application.

### 4.4 Processing speed and failures

**Fig. 4.**
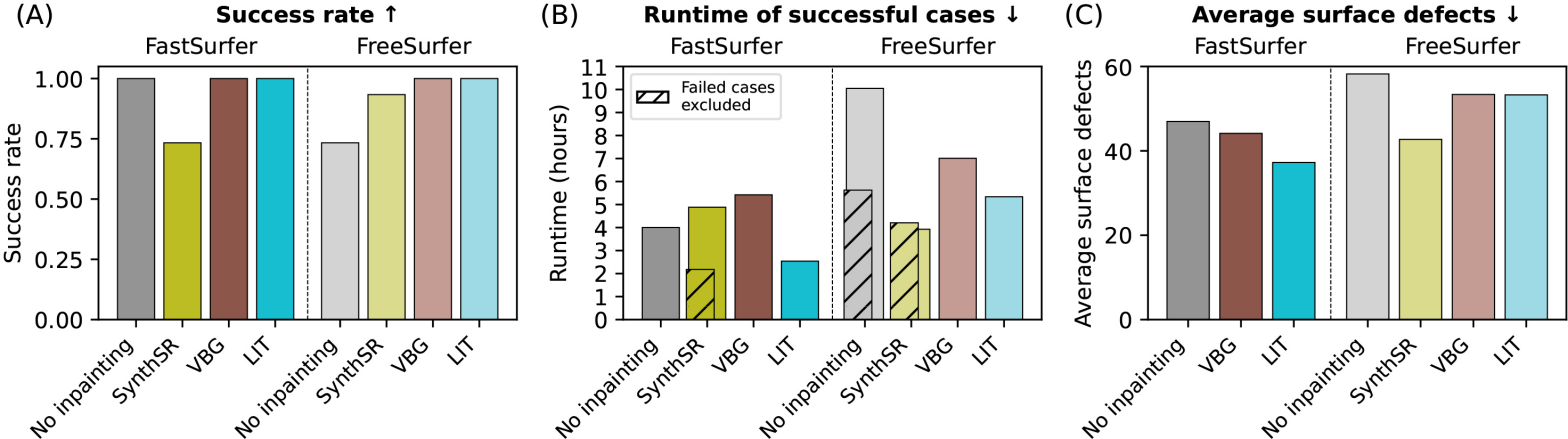
From left to right: The success rate of method (fraction of segmentations and surfaces generated within 24 hours), runtime on a desktop workstation, number of surface defects before fixing of surface topology. Subfigures A+B are based on 15 cases of the patient dataset, while Subfigure C is based on 100 cases of the synthetic lesion dataset.

**Appendix Fig. A2.**
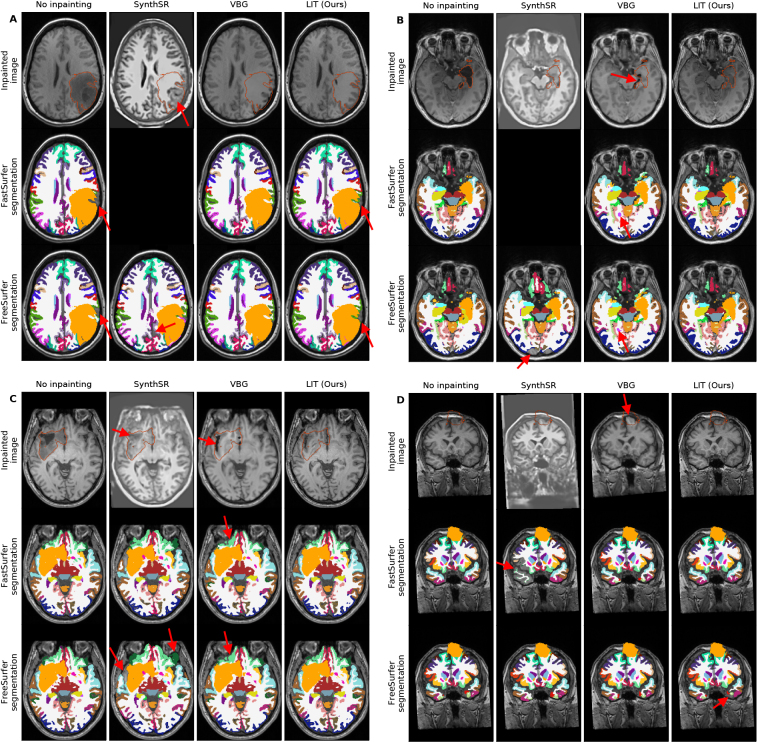
Qualitative comparison of whole-brain segmentations for all method combinations on cases randomly selected from the UPENN (A), UKB (B), preoperative BTC (C), and postoperative BTC (D) dataset. Empty spots for SynthSR + FastSurfer represent method failures. The shown slices are located at the center of the inpainting mask. Red arrows point at obvious inpainting and segmentation flaws.

We evaluate success rate and overall method runtime on a subset of 10 cases of the patient dataset. Here, we test all combinations of the inpainting tools (SynthSR, VBG, LIT) with FastSurfer and FreeSurfer. We show the success rates in Figure 4A. In the first evaluation scenario (using FastSurfer), only the SynthSR variant fails (4/15 cases). We hypothesize that the segmentation failures of SynthSR + FastSurfer stem from an atypical intensity distribution incompatible with FastSurfer (see Appendix Fig. A2). We conclude that the two methods are incompatible and exclude this combination from other evaluations. For the second scenario (using FreeSurfer), failures occur when using vanilla FreeSurfer (no inpainting) (4/15) and SynthSR for inpainting (1/15). For later comparisons (Section 4.6), we additionally run the two most promising methods VBG and LIT on the whole patient dataset, where none of them fails for any of the 270 cases.

We also show the average time to fully process an image including inpainting, segmentation, and surface creation in Figure 4B. For the FastSurfer combinations, LIT is fastest, followed by vanilla FastSurfer (no inpainting), VBG, and SynthSR. The long processing time of SynthSR is driven by the failure cases, but even when looking only at the 11 successful cases, it is still only slightly faster than LIT (average runtime on all 15 cases). VBG has an overall runtime of 5 hours, taking more than twice as long as LIT. Especially for the FastSurfer toolbox, the overhead of the slow template-based VBG inpainting is disproportionate to its fast segmentation and surface reconstruction. For methods run in conjunction with FreeSurfer, SynthSR generates outputs the fastest, followed by LIT, VBG, and vanilla FreeSurfer (no inpainting). In this case, crashes decrease the runtime for SynthSR, as these crashes seem to have happened early in FreeSurfer processing. Vanilla FreeSurfer has a slightly longer runtime on successful cases than LIT (on all cases)—showing that additional inpainting time is largely compensated in LIT by faster downstream processing, for example, during surface creation.

### 4.5 Surface defects

We assess the quality of surfaces by counting the defects prior to topology fixing in FastSurfer and FreeSurfer on the synthetic glioblastoma dataset and show the results in Figure 4C. For the FastSurfer scenario, LIT inpainting results in the fewest surface defects, followed by VBG and the vanilla FastSurfer baseline (no inpainting). For method combinations with FreeSurfer, SynthSR produces the least surface defects, followed by LIT and VBG with similar number of defects. The FreeSurfer baseline produces the most defects overall. The reduction in surface defects as a result of the inpainting as a prepossessing step causes FreeSurfer and FastSurfer to run faster (shown in Section 4.4)—fewer surface defects require less computationally expensive topology fixing.

### 4.6 Comparison on patient data

We choose the two previously best performing methods VBG and LIT and let a domain expert compare both methods with FreeSurfer and FastSurfer segmentations using the previously discussed protocol (see Section 3.6.4).

For the FastSurfer processing, LIT inpainting produces better whole-brain segmentations in 93% of decidable cases (in 18% of all cases no decision was possible, see Table 2). The same trend holds true for the FreeSurfer segmentations, where LIT is chosen better in 91% of decidable cases (10% no decision). A review of the comments reveals that if no method was decided to be superior, this was most often due to low image quality, high similarity between segmentations, or inaccurate lesion masks.

**Table 2.** Results of manual quality rating on patient data.

Method	N	VBG+FastSurfer	LIT+FastSurfer
Winning Method	50	3	38
Failures (random)	23	17%	4%
Failures (high diff.)	27	37%	0%

Method	N	VBG+FreeSurfer	LIT+FreeSurfer
Winning Method	50	4	41
Failures (random)	27	15%	0%
Failures (high diff.)	23	70%	4%

In the first row, we compare VBG and our method (LIT) on randomly selected cases and cases selected according to large difference in segmentation maps (high diff.). A blinded rater selected the method with better segmentation maps as “Winning Method.” The rater also annotates segmentation failures shown separately for high diff. cases and others (random). Ratings for VBG and LIT differ significantly in direct method comparison and failures on high difference cases (p<0.005), but not for failures on the random cases.

On the second, failure rating task, FastSurfer in combination with LIT, shows only one failed segmentation for random samples (4%) and no failure (0%) on the high difference set. VBG on the other hand fails 4 times (17%) on random samples and 10 times (37%) on the more challenging high difference cases. For the FreeSurfer-based comparison, LIT fails for no cases (0%) on the random set and for one case on the high difference set (4%), while VBG fails for 4 (15%) and 16 cases (70%), respectively. Overall LIT inpainting is superior combined with both FreeSurfer and FastSurfer methods, providing more accurate segmentations maps in direct comparison and also producing less failures. This is especially true for the high difference set, where low agreement is likely caused by frequent VBG failures and its downstream effects.

### 4.7 Qualitative results

**Fig. 5.**
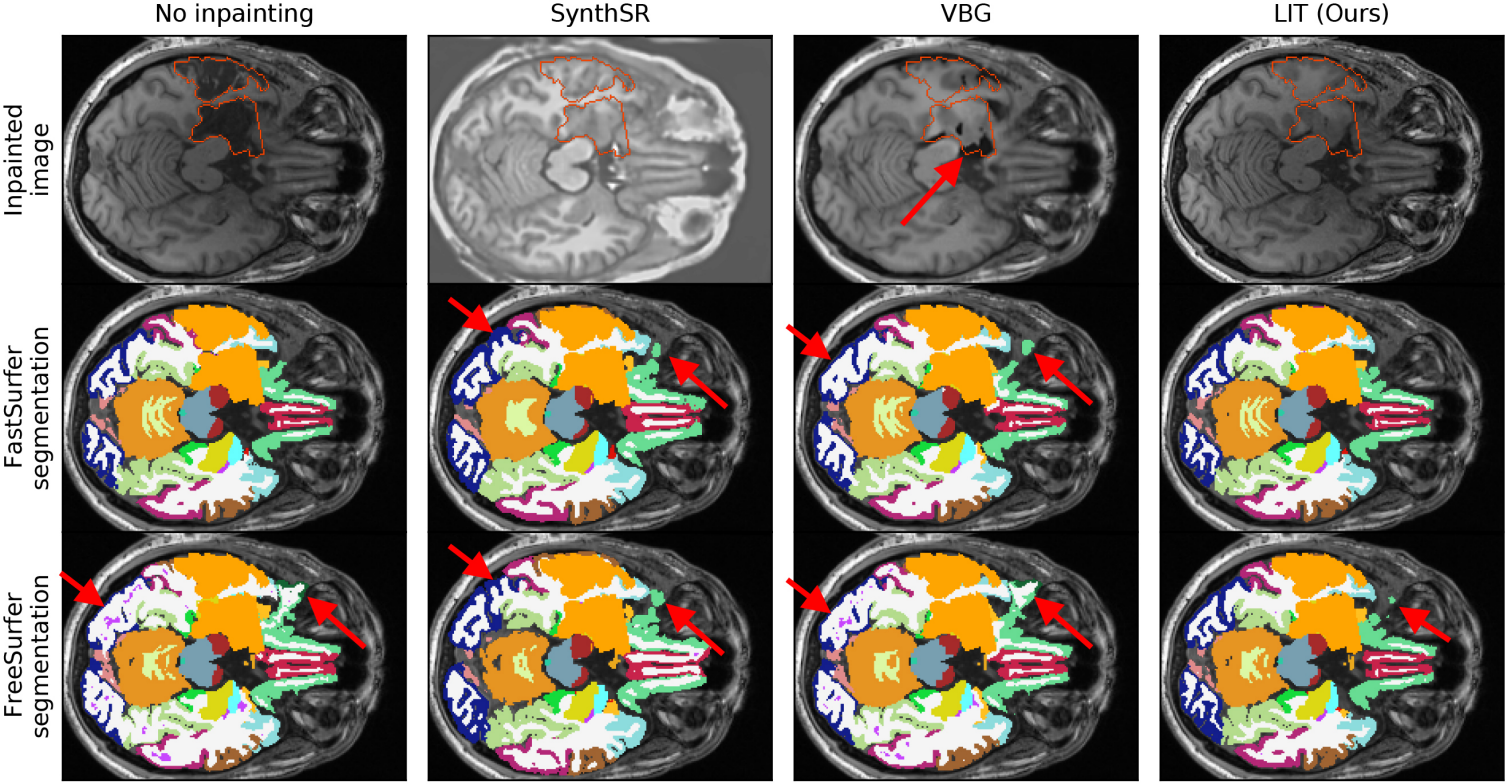
Qualitative comparison of whole-brain segmentations for all method combinations on a representative case from the UKB dataset. For randomly selected and difficult cases, see Appendix Figures A2 and A3, respectively. The shown slices are located at the center of the lesion. Red arrows indicate inpainting and segmentation flaws.

In Figure 5 we show a representative case, where a cavity was caused by a transsylvian hippocampal resection. On the top left we show the original image (no inpainting). The inpainting of SynthSR fills the lesion area with plausible tissue, however, we can see that the image contrast is changed everywhere, leading to gray matter, that appears thicker than in the original and a general loss of detail in the image. VBG fills parts of the cortical area with plausible tissue, but produces sharp edges and cavities in the subcortical structures (red arrow). Our LIT inpainting fills the area with plausible tissue and continues structures outside of the mask.

In the resulting FastSurfer segmentations, we observe that FastSurfer without inpainting generates visually plausible results. SynthSRs contrast adjustment, however, leads to generally enlarged gray matter volume for this case. Additionally, a fold with strong over-segmentation was introduced (posterior red arrow). At the front of the gray matter is also over-segmented (anterior red arrow). The FastSurfer segmentation based on VBG inpainting causes gray matter under-segmentation in the posterior regions (e.g., red arrow) and over-segmentation in the anterior region (red arrow). For our LIT inpainting, the posterior regions are segmented as accurate as with the vanilla FastSurfer. In the anterior region, previously challenging for other methods, our method performs as expected, resulting in more accurate segmentation.

For FreeSurfer, the baseline without inpainting contains extremely under-segmented gray matter (posterior red arrow) and erroneous hypointensities. It also contains extreme over-segmentation in the challenging anterior region (red arrow). SynthSR slightly improves the segmentation in the anterior region, but causes the same over-segmentation in the posterior region, as previously seen with FastSurfer. The FreeSurfer segmentation map based on VBG inpainting shows the same issues as the FreeSurfer segmentation without inpainting. Our LIT inpainting results in accurate segmentation of the posterior regions, and only slight over-segmentation in the anterior region (red arrow).

**Appendix Fig. A3.**
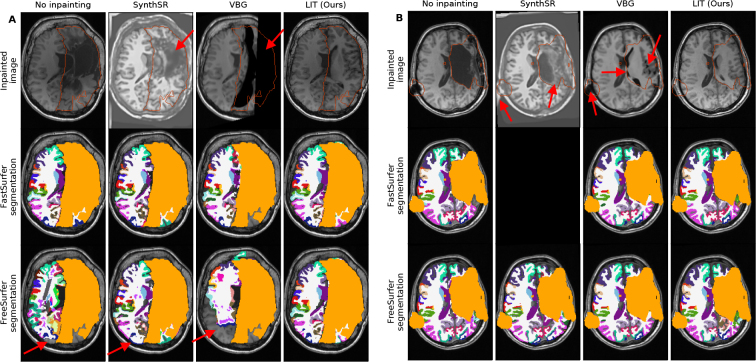
Qualitative comparison of two very challenging cases. Shown are whole-brain segmentations for all method combinations. Empty spots for SynthSR + FastSurfer represent method failures. The shown slices are located at the center of the inpainting mask. Red arrows point at obvious inpainting and segmentation flaws.

**Appendix Fig. A5:**
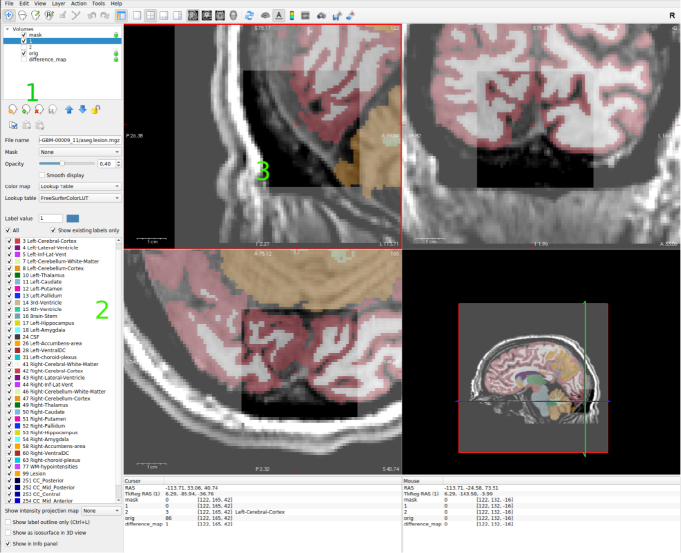
Screenshot from the window for the comparison of whole-brain segmentations. The green numbers denote (1) the area in Freeview to manage loading volumes, showing segmentations as 1/2, (2) an overview over some of the segmentation in the label map, (3) the focus area, which is an area of low inter-method agreement pointed out to the rater.

We show further qualitative results in the Appendix B: Figures A2 (randomly selected cases) and A3 (challenging cases). These cases outline that our method is the only method to modify only the given replacement area (Appendix Fig. A2.A), accurately inpaints non-brain tissue and skull (Appendix Figs. A2.D and A3.B), and handles very large abnormalities well (Appendix Fig. A3.A).

### 4.8 Cortical thickness estimates before and after surgery

**Fig. 6.**
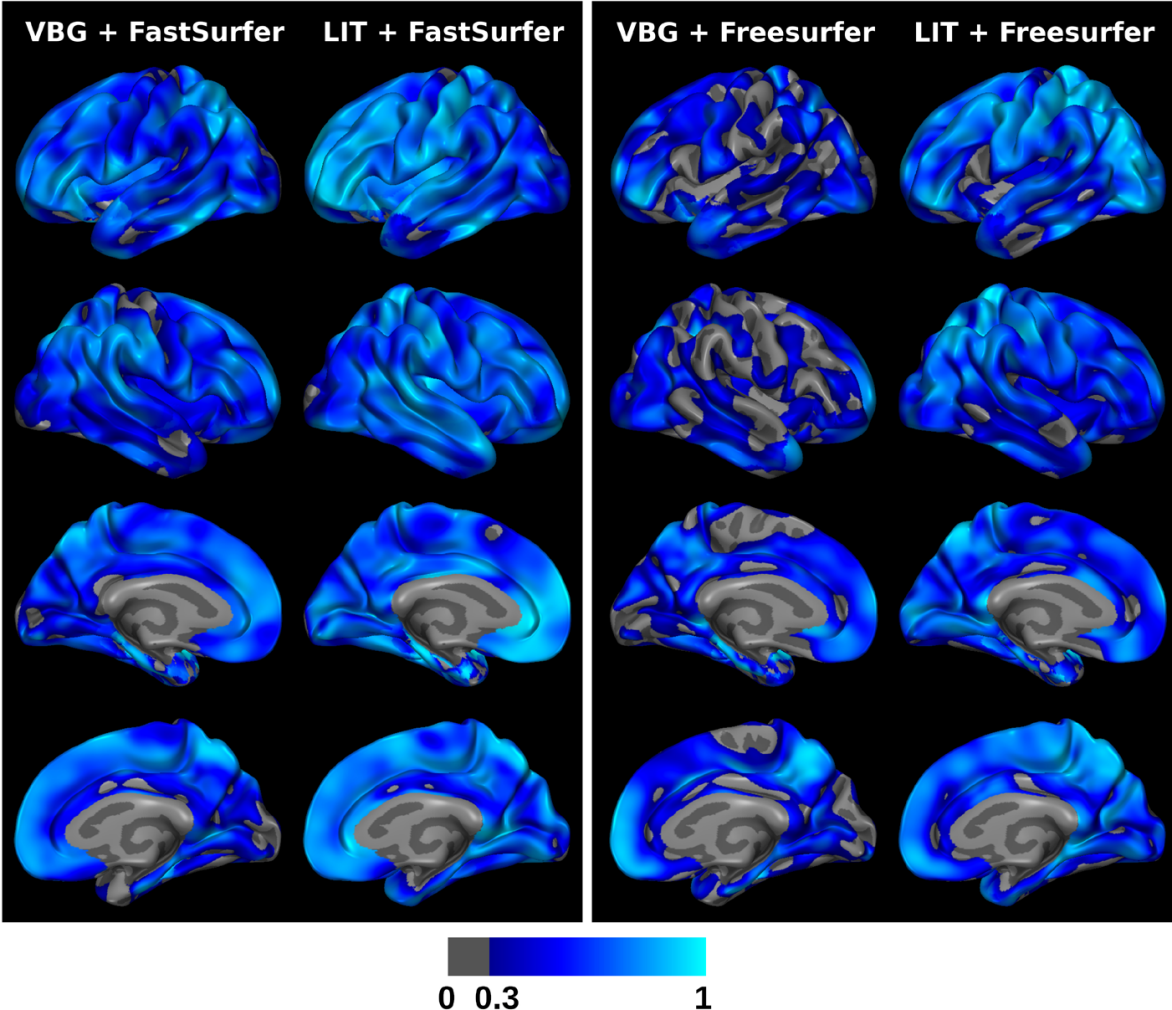
Comparisons of intraclass correlation coefficient (ICC) values for the cortical thickness of patients before and after temporomesial resection. Each column has different views of the semi-inflated template brain surface, where lighter shades of blue indicate a higher ICC and higher reproducibility of thickness estimates, when using inpainting for lesion filling.

We evaluate the consistency of cortical thickness estimates on MRI from patients before and after undergoing temporomesial resection surgery for VBG and LIT. We show the intraclass correlation coefficients on the surface of a template brain for all methods in Figure 6. As it is reasonable to assume that the cortical thickness does not change between pre- and post-operative scans, the ICC of cortical thickness reflects how robust the cortical thickness analysis is when introducing lesion inpainting. In a best-case scenario, the only difference between cortical thickness estimates are test–retest effects.

For the FastSurfer scenario, we observe generally higher ICC values with LIT than with VBG specifically in frontal and postcentral regions. For the FreeSurfer scenario, we observe the same trend, with the LIT variant also having ICC values close to 1 in several regions (e.g., superior frontal, postcentral, parietal, and occipital). VBG has large areas with very low numbers below 0.3 though. When comparing FastSurfer with FreeSurfer, we see generally higher ICC for FastSurfer, which is consistent with previously reported test–retest results (Henschel et al., 2020).

## 5 Discussion

In this work, we introduce FastSurfer-LIT, a whole-brain segmentation and surface reconstruction pipeline for structural MRI with lesions and cavities. As shown in the ablation study, our extension to the latent DDPM architecture and inference improves segmentation quality. We leverage this inpainting quality to improve whole-brain segmentation and surface reconstruction in the presence of lesions. Our pipeline outperforms state-of-the-art methods in four evaluations ranging from experiments on MRI with synthetic lesions, expert ratings on our highly heterogeneous, real-world patient dataset with 14 different types of lesions, and to a consistency of cortical thickness analysis of patient MRI. The variety of lesions and their size in our datasets highlights the robustness and breadth of application of our method (see Appendix Fig. A3 for challenging cases). While competing methods were previously limited to 1 mm voxel resolution, our method can natively inpaint sub-millimeter MRI, which enables analysis for images with higher level of detail. Finally, with 2.5 hours of runtime on average for joint segmentation and cortical reconstruction, or approximately 30 minutes for whole-brain segmentation only, FastSurfer-LIT is an efficient tool for morphometric analysis. While generally much faster than the previous template-based approach (VBG), its speed does not stem from fast inpainting alone (as the iterative reverse diffusion is slower than, for example, the single shot SynthSR), but also from robust and accurate inpainting that enables the segmentation and surface reconstruction to be faster, for example, because of fewer topological surface defects.

The superior performance on challenging cases with either large or unusual lesions is unique to our method, because it is the only one that is designed to be unaffected by the shape or appearance of the lesion. Furthermore, our method guarantees to leave the image outside of the mask completely unchanged during the inpainting process, which is reflected in higher quality segmentations in areas outside of the lesion mask. Finally, our learned brain model allows LIT to generate tissue that is completely individualized to the target brain, leading to more plausible inpaintings than template-based approaches (see Appendix Fig. A2). A disadvantage of individualized inpainting is that when masks do not cover a whole cavity, the unmasked part might be expanded into the lesion mask to create a plausible inpainting—for such cases we recommend dilating the lesion mask. Fortunately, the expansion of tumors or other distinct pathologies is unlikely, since these cases are not seen during training and are, therefore, not part of the learned brain model.

By integrating FastSurfer-LIT into the FastSurfer project (https://github.com/Deep-MI/FastSurfer), we hope to accelerate research on the impact of interventions and disease (e.g., radiotherapy, surgery, glioblastoma) on overall brain health. FastSurfer-LIT could grant new insights into cortical reorganization (Zhang et al., 2022), with higher resolution data, more accurate cortical reconstruction, and the ability to exclude only the lesion area, instead of limiting the analysis to areas where all patients are lesion free. Besides accelerating and improving research, this work can also contribute to fairness and accessibility of personalized medicine (e.g., personalized structural connectomics (Imms et al., 2023)), which can be made available for individuals with abnormal brain structures.

As segmentation of abnormal areas is out of the scope of this work, masks have to be generated manually or by one of the many available segmentation methods for lesions (as done previously by Zhang et al. (2022)). In the future, FastSurfer-LIT may be extended with more general anomaly detection (Wolleb et al., 2022) to make the pipeline fully automatic. Alternatively researchers could also generously mask tissue that is not of interest (e.g., one entire hemisphere, similar to Appendix Fig. A3.A) to make sure lesion tissue is excluded, which removes the requirement for accurate segmentation. Since LIT can run independently from the downstream segmentation or surface reconstruction steps of the pipeline, it can easily be combined with other neuroimaging tools. In our evaluation, we focus on segmentation and surface reconstruction with challenging data. We expect LIT to also enable use of other neuroimage analysis software with lesions, such as structural sub-segmentation (Estrada et al., 2023; Faber et al., 2022) or subject-to-subject and subject-to-atlas registration (previously demonstrated by SynthSR (Baheti et al., 2021; Iglesias et al., 2023)). Therefore, we release both, a standalone version of the tool (https://github.com/Deep-MI/LIT/) and also an integration into the FastSurfer toolbox (https://github.com/Deep-MI/FastSurfer/).

Overall, we introduce FastSurfer-LIT, an accurate pipeline for automated neuroimage analysis of brains with cavities, tumors, and other lesions of any size. The pipeline works on multiple resolutions, enabling sub-millimeter analyses for the first time and outperforms previous approaches shown by rigorous analysis.

## Figures and Tables

**Appendix Fig. A4:**
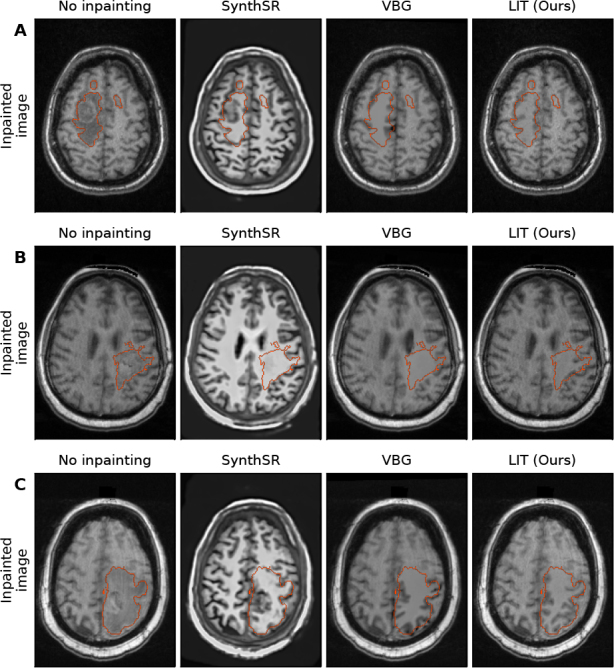
Qualitative comparison of cases with reduced image quality due to motion artifacts. Shown are the original images and inpainted images produced by SynthSR, VBG, and LIT. The shown slices are located at the center of the inpainting mask.

## Data Availability

The source code for FastSurfer-LIT will be integrated into the FastSurfer pipeline at https://github.com/Deep-MI/FastSurfer and available as standalone inpainting tool at https://github.com/Deep-MI/LIT. The rating tool used for the user study is available at https://github.com/Deep-MI/segmentation_labeling. All MRI datasets of the no-lesion dataset are publicly available and references to the open-source repositories are provided in Appendix Figure A3 of the FastSurferVINN paper (Henschel et al., 2022). The BTC dataset (Aerts & Marinazzo, 2018; Aerts et al., 2018, 2020) is available at https://openneuro.org/datasets/ds001226/versions/00001, the UPENN-GBM dataset (Bakas et al., 2021, 2022; Clark et al., 2013) is available at the Cancer Imaging Archive https://wiki.cancerimagingarchive.net/pages/viewpage.action?pageId=70225642 and the MSSEG dataset (Commowick et al., 2018, 2021) is available at SHANOIR https://shanoir.irisa.fr/shanoir-ng/welcome. The UKB data are not publicly available due to their containing information that could compromise the privacy of research participants. Data of the Rhineland Study are not publicly available because of data protection regulations. However, access can be provided to scientists in accordance with the Rhineland Study’s Data Use and Access Policy. Requests to access the data should be directed to Dr. Monique Breteler at RS-DUAC@dzne.de.

## Footnotes

* Data of the 2016 MSSEG challenge dataset were generated by neurologists in OFSEP, the French MS registry (Vukusic et al., 2020). They collect clinical data prospectively in the European Database for MS (EDMUS) software (Confavreux et al., 1992). MRI of patients were provided as part of a care protocol. Nominative data are deleted from MRI before transfer and storage on the Shanoir platform (“SHANOIR SharingNeurOImagingResources”, n.d.).

## Appendix A

### A. Inpainting Data Flow

In this work, we train the DDPM U-Net (Pinaya et al., 2022, 2023; Rombach et al., 2022) on lesion-free images. During the inference, we condition the network by continuously replacing the area outside of the lesion mask with a noised portion of the original image. We visualize this process in Appendix Figure A1 and also point to previous publications in the field of computer vision for in-depth background (Dhariwal & Nichol, 2021; Lugmayr et al., 2022). The implementation details for the neural network and its training can be found in the Open Source repository: https://github.com/Deep-MI/LIT.

## Appendix B

### B. Qualitative Inpainting Results

Some quality metrics for inpainting struggle to capture the complexity of the task. Inpainting regions should seamlessly match the surrounding area (including structural integrity, noise and intensity levels) and also produce plausible brain structures that depend on the position of the inpainted region, and also on the appearance of the rest of the brain. For a qualitative comparison, we show additional inpainted images with segmentation maps in Appendix Figures A2–A4. First we show a randomly selected case from each of the clinical dataset in Appendix Figure A2. In the inpainted images on the top rows, we observe that the competing methods can modify the image outside of the inpainting mask, which is undesirable and avoided by LIT. SynthSR struggles especially with large lesions (A.2.A) and VBG with complex shapes (A.2.B) and non-brain tissue (A.2.D). In Appendix Figure A3, we show extremely challenging cases, where the competing methods do not produce (meaningful) inpaintings. Meanwhile our method performs as expected. Finally, in Appendix Figure A4, we show that our inpainting network also works in the presence of motion artifacts and low signal-to-noise ratio. While the inpainting can enable use of certain tools, we recommend excluding cases with extreme motion artifacts from analysis with FreeSurfer and FastSurfer, since artifacts are known to bias downstream analysis tasks (Reuter et al., 2015).

## Appendix C

### C. Rating Procedure

Judging and comparing the quality of whole-brain segmentation can be a very time consuming process. To compare two methods that are giving segmentation of similar quality, raters would typically go through the whole-brain volume and inspect all the different areas. Then they would often identify one or more areas, which show large differences and finally base their judgment on these areas of interest. Based on these observations, we develop an open-source tool and streamline the rating process for the comparison of segmentation maps (shown in Appendix Fig. A5, available on GitHub (https://github.com/Deep-MI/segmentation_labeling). The goal is to create reliable labels while reducing the required inspection time. This allows us to include more images into this analysis than previous methods (Radwan et al., 2021). We aim to (i) provide a responsive user interface and (ii) focus the attention of raters. Our rating tool is based on FreeSurfer’s freeview, which is an MR image viewer well known in the community and often does not require additional training of raters. We open and close it from a software package that manages the rating process. To speed up waiting times, all cases are pre-processed and pre-loaded. In addition to the original MRI and the two to-be-compared segmentations maps, we also show a *focus area* and a *difference map*, which can aid the blinded raters by drawing their attention to differences between methods. The *focus area* highlights a suggested area for inspection and decision making. The *difference map* can be switched on to indicate where the segmentation maps disagree, and even spot subtle differences.
